# Maporal Hantavirus Causes Mild Pathology in Deer Mice (*Peromyscus maniculatus*)

**DOI:** 10.3390/v8100286

**Published:** 2016-10-18

**Authors:** Amanda McGuire, Kaitlyn Miedema, Joseph R. Fauver, Amber Rico, Tawfik Aboellail, Sandra L. Quackenbush, Ann Hawkinson, Tony Schountz

**Affiliations:** 1 Arthropod-Borne and Infectious Diseases Laboratory, Department of Microbiology, Immunology and Pathology, College of Veterinary Medicine and Biomedical Sciences, Colorado State University, Fort Collins, CO 80523, USA; amanda.mcguire@colostate.edu (A.M.); kaitlyn.miedema@colostate.edu (K.M.); joseph.fauver@colostate.edu (J.R.F.); tawfik.aboellail@colostate.edu (T.A.); 2 School of Veterinary Medicine and Biomedical Sciences, University of Nebraska, Lincoln, NE 68583, USA; arico3@unl.edu; 3 Department of Microbiology, Immunology and Pathology, College of Veterinary Medicine and Biomedical Sciences, Colorado State University, Fort Collins, CO 80523, USA; sandra.quackenbush@colostate.edu; 4 School of Biological Sciences, University of Northern Colorado, Greeley, CO 80639, USA; ann.hawkinson@unco.edu

**Keywords:** hantavirus, Maporal virus, deer mice, reservoir, zoonoses

## Abstract

Rodent-borne hantaviruses can cause two human diseases with many pathological similarities: hantavirus cardiopulmonary syndrome (HCPS) in the western hemisphere and hemorrhagic fever with renal syndrome in the eastern hemisphere. Each virus is hosted by specific reservoir species without conspicuous disease. HCPS-causing hantaviruses require animal biosafety level-4 (ABSL-4) containment, which substantially limits experimental research of interactions between the viruses and their reservoir hosts. Maporal virus (MAPV) is a South American hantavirus not known to cause disease in humans, thus it can be manipulated under ABSL-3 conditions. The aim of this study was to develop an ABSL-3 hantavirus infection model using the deer mouse (*Peromyscus maniculatus*), the natural reservoir host of Sin Nombre virus (SNV), and a virus that is pathogenic in another animal model to examine immune response of a reservoir host species. Deer mice were inoculated with MAPV, and viral RNA was detected in several organs of all deer mice during the 56 day experiment. Infected animals generated both nucleocapsid-specific and neutralizing antibodies. Histopathological lesions were minimal to mild with the peak of the lesions detected at 7–14 days postinfection, mainly in the lungs, heart, and liver. Low to modest levels of cytokine gene expression were detected in spleens and lungs of infected deer mice, and deer mouse primary pulmonary cells generated with endothelial cell growth factors were susceptible to MAPV with viral RNA accumulating in the cellular fraction compared to infected Vero cells. Most features resembled that of SNV infection of deer mice, suggesting this model may be an ABSL-3 surrogate for studying the host response of a New World hantavirus reservoir.

## 1. Introduction

Hantaviruses are negative-stranded, trisegmented members of the *Bunyaviridae* family that are hosted by rodents, insectivores, or bats [1,2]. Several pathogenic hantaviruses, all of which are hosted by rodents, can cause hemorrhagic fever with renal syndrome (HFRS) in Eurasia, or hantavirus cardiopulmonary syndrome (HCPS) in the Americas [3,4]. Although the principal target organs of these diseases differ, both infect the endothelium and share many pathologic similarities that lead to fluid loss and electrolyte imbalances, suggesting the underlying disease mechanisms are similar [5,6]. Some evidence suggests a role for the immune response in pathogenesis of hantavirus disease [7,8,9], but a role for lymphocytes is unclear [10,11]. The principal cellular target of hantaviruses are vascular endothelial cells but without conspicuous effects on those cells, although vascular leakage is a prominent feature of hantavirus disease [12].

Deer mice (*Peromyscus maniculatus*) are the principal reservoir hosts of Sin Nombre virus (SNV) [13,14,15], the etiologic agent of most HCPS cases in North America [1,4]. Inoculation of deer mice with SNV results in persistent infection without signs of clinical disease, and neutralizing antibodies appear after three weeks; however, they are insufficient for clearing virus [16,17,18]. Early immune markers suggest a subtle Th1/Th2 response, followed by signatures of regulatory T cell responses that may impair cytotoxic T cell activities and contribute to persistent infection [18,19,20].

Experimental infection of deer mice with Andes virus (ANDV) results in an apathogenic infection but, in contrast, a robust immune response occurs that is associated with clearance within several weeks [20,21]. The immune response is characterized by prominent expression of Th2 and T follicular helper cell genes, STAT1 (signal transducer and activator of transcription 1) phosphorylation, interleukin (IL)-4 pathway genes and, to a lesser extent, Th1 genes, and earlier seroconversion suggesting a rapid maturation of the IgG response.

Human pathogenic New World hantaviruses, such as SNV and ANDV, require animal biosafety level-4 (ABSL-4) containment [22]. Maporal virus (MAPV) is closely related to ANDV and was first isolated from a fulvous pygmy rice rat (*Oligoryzomys fulvescens*) captured in Venezuela [23]; however, subsequent work identified the delicate pygmy rice rat (*O. delicatus*) as the principal reservoir host [24]. Similar to ANDV, and unlike SNV, MAPV causes an HCPS-like disease in experimentally infected Syrian hamsters (*Mesocricetus auratus*) [25]. Because MAPV is not known to cause disease in humans, it can be used in experimental infections with ABSL-3 precautions.

We were interested in determining if deer mice are susceptible to MAPV and, if so, whether they developed clinical disease (like hamsters), cleared infection (like ANDV), or remained persistently infected (like SNV). We inoculated deer mice with MAPV and detected virus in the lungs 2 days later and until the termination of the experiment on day 56. MAPV-specific antibodies were detected in deer mice as early as 14 days postinfection (PI). Viral RNA was also detected in the lungs, heart, spleen, kidney, liver, and salivary glands 14 days after inoculation. Although no conspicuous signs of disease were observed, we found indications of mild pathology in several organs upon histological examination. We also found that deer mouse pulmonary cells propagated in endothelial cell medium were susceptible to MAPV infection and that virus may transmit directly to adjacent cells. Many of these characteristics more closely resemble SNV infection of deer mice than ANDV infection. Thus, MAPV infection of deer mice offers a suitable ABSL-3 system in which to study the host response of a reservoir of a New World hantavirus with pathogenic potential.

## 2. Materials and Methods

### 2.1. Ethics Statement

All procedures using deer mice were in compliance with the U.S. Animal Welfare Act and approved by the Colorado State University institutional animal care and use committee (protocol 13-4377A) and performed by following the guidelines of the Association for Assessment and Accreditation of Laboratory Animal Care, International (AAALAC), by certified staff in an AAALAC-approved facility.

### 2.2. Experimental Infections

Deer mice of both sexes and aged 8–16 weeks were used for this study. All procedures with MAPV were approved by the Colorado State University Institutional Biosafety Committee. MAPV HV 97021050 (kindly provided by D. Safronetz, Rocky Mountain Laboratories, NIAID) was propagated in Vero E6 cells (ATCC CRL-1586). In the initial susceptibility testing, 10 deer mice (5 males, 5 females) were subcutaneously inoculated in the left hindquarters with 10^4^ 50% tissue culture infectious dose (TCID_50_) MAPV, and two deer mice (1 male, 1 female) were sham-inoculated with sterile PBS. On days 2, 4, 7, 14, and 56, one male and one female were euthanized for blood collection, necropsy, and tissue retrieval. Tissues for RNA extraction were immediately placed in liquid nitrogen, then stored at −80 °C, whereas tissues for histopathology were placed in 10% buffered formalin.

In a second experiment, five male deer mice were inoculated as described in the susceptibility experiment, and were euthanized 14 days after infection for examination of viral infection by PCR and immunohistochemistry (IHC), immune gene expression, and for histopathology of lungs, hearts, spleens, kidneys, livers, and salivary glands.

### 2.3. Tissue Processing

Tissues were prepared for histopathology and immunochemistry as previously described [18]. Tissues were sectioned and stained with hematoxylin–eosin with at least 2 sections examined blindly per tissue. Both heart ventricles were sectioned, including the septum. Five uninfected control animals (day 14) were also examined. Scoring was 0–3 and performed under high-power (40×) microscopic fields (HPMF) for no lesions (−), presence of fewer than 5 necrotic/degenerate (N/D) cells and 5–10 inflammatory cells (IC) (+/−), 5–10 and N/D 10–25 IC (+), and >10 N/D and >25 IC (++). For IHC, polyclonal rabbit anti-SNV nucleocapsid antiserum (kindly provided by B. Hjelle, University of New Mexico Medical Center) that we have previously used with SNV-infected deer mice [18] and that is cross-reactive with ANDV [21] was used to detect viral antigen.

Virus isolation was attempted from tissues by preparing homogenates with stainless steel beads in 5% FBS–DMEM (TissueLyser LT, Qiagen, Valencia, CA, USA). Samples were homogenized 2 × 5 min at 2 Hz, and then centrifuged at 3000 rpm for 5 min at 4 °C. Four hundred microliters of the supernatant was added to 500 μL 5% FBS–DMEM and filtered through 0.2 μm Acrodisc filter and 100 μL of filtrate inoculated onto confluent Vero E6 cells in 24 well plates. Medium was harvested on day 7 and RNA was extracted from supernatants (QIAamp Viral RNA Mini kit, Qiagen) according to manufacturer’s instructions. One-step PCR was performed and sample run on 1% agarose gels.

### 2.4. ELISA

A previously described protein-A/G–HRP (horseradish peroxidase) enzyme immunoassay was used to detect IgG [26]. Briefly, a truncated SNV nucleocapsid antigen containing a highly conserved epitope [27] was diluted to 1 μg/mL in PBS (pH 7.2) and 100 μL added to wells of a 96-well polyvinyl chloride (PVC) microtiter plate (BD Falcon, Franklin Lakes, NJ, USA) that was incubated overnight at 4 °C. The next day, the plate was washed with PBS, then blocked (SuperBlock T20, Thermo Scientific, Waltham, MA, USA). After washing, serum samples were diluted to 1:100 in PBS, then further diluted (log_2_) to determine endpoint titers. Sera were incubated for 1 h at room temperature, then washed 4× in PBS-0.5% TWEEN20. Protein-A/G–HRP (32490, Thermo Scientific) conjugate (1:5000) was incubated for 1 h at room temperature, followed by washing and the addition of ABTS substrate (50-62-00, KPL, Gaithersburg, MD, USA) for 15 min. Absorbances at 405 nm were recorded (model 450, Bio-Rad, Hercules, CA, USA). Samples with readings 0.200 above the mean of the negative control serum samples were considered positive. Endpoint titers were calculated as the reciprocal of the greatest dilution that yielded a positive result, and the geometric means (GMT) and standard deviations (SD) plotted.

### 2.5. Serum Neutralization Assay

Sera from the infected deer mice were diluted 1:20 in 2% FBS–DMEM, then diluted log_2_ in final volumes of 100 μL [18]. MAPV was diluted to 10^3^ TCID_50_/mL and 100 μL added to the serial serum dilutions (1:2 dilution) and incubated for 1 h at room temperature. The 200 μL volumes were then pipetted onto Vero E6 cells monolayered on cover slips in 24-well plates and incubated for 1 h at 37 °C, followed by the addition of 800 μL of 2% FBS–DMEM. Plates were incubated for 7 days, then fixed in 2% buffered formalin, followed by detection of viral antigen using rabbit anti-SNV nucleocapsid-specific antiserum. Goat anti-rabbit IgG–FITC conjugate was incubated for 2 h. Coverslips were mounted on microscope slides with DAPI (ProLong Gold, Life Technologies, Foster City, CA, USA) for visualization by fluorescent microscopy. The reciprocal of the greatest dilution of serum that blocked infection was considered the endpoint titer.

### 2.6. Detection of Viral RNA in Tissues

A qualitative reverse-transcription PCR assay was used to detect viral RNA. Total RNA was extracted (RNEasy kit, Qiagen) and used for PCR (OneStep RT-PCR kit, Qiagen). Primers used were from the MAPV S segment, 5′-AGCTGTGATGAGCAACCTCC-3′ (forward) and 5′-CAATTGGCACAAGCCCGAAA-3′ (reverse) with 95 °C melting (30″), 58 °C annealing (30″), and 72 °C extension (60″) for 35 cycles. Amplicons were resolved on agarose gels and also sequenced to verify MAPV.

### 2.7. Cytokine PCR

Real-time PCR for cytokine and chemokine gene expression in lungs and spleens was performed as previously described [18,20,28]. Briefly, total RNA was reverse transcribed to synthesize cDNA for amplification of transcripts (Two-Step Go-Taq SYBR Green, Promega, Madison, WI, USA) to determine relative fold changes using the ΔΔCt method [29]. Gapdh (glyceraldehyde-3-phosphate dehydrogenase) was used in duplicate as within-sample normalization control (ΔCt), followed by comparison of the same gene between infected deer mice and the mean ΔCt of two uninfected control RNA samples (ΔΔCt). Fold-changes of each gene from each animal were determined and 95% confidence intervals calculated to provide margins of error for each gene. Error bars that do not overlap are considered statistically significant using this method. Heat maps and hierarchical clusters were generated with R statistical software (http://www.r-project.org/) using the heatmap.2 (gplots) package [30].

### 2.8. Generation of Deer Mouse Pulmonary Microvascular Endothelial Cells

Seven day old deer mice were euthanized for retrieval of lungs and isolation of pulmonary microvascular endothelial cells (PMVEC) using a modified strategy [31]. Lungs were aseptically minced with a sterile scalpel and incubated with a collagenase/elastase cocktail (E7885, C1639, Sigma, St. Louis, CA, USA) for 1 h at 4 °C with gentle tumbling on an orbital mixer. Digested tissue suspension was then aspirated into a 30 mL syringe and slowly expelled through a 20 gauge needle to separate tissue clumps into a single cell suspension. This was repeated 12 times to ensure maximum separation. Fifteen milliliters of 20% FBS–DMEM was added to stop enzymatic activities. Particulates were allowed to briefly settle and the cell suspension was collected and washed in 0.1% BSA DPBS, and then resuspended in 3 mL of endothelial cell medium (ECM; VascuLife EnGS-Mv Life Factors kit, 10% ΔH FBS, LL-0004, Lifeline Cell Technologies, Oceanside, CA, USA). T-75 flasks were coated with bovine skin gelatin (G1393, Sigma) and allowed to dry. Cells were plated in gelatin-coated T-75 flasks at 2.1 × 10^6^ cells in 10 mL of 10% FBS–ECM. Because of their rapid replication, cells were passaged 1:20 at 4 day intervals and frozen stocks prepared.

### 2.9. PMVEC Infections

For infection experiments, cells were plated onto sterile glass coverslips in 24-well plates. MAPV was diluted in 2% FBS Ham’s F12 medium and 200 μL containing 0.1 MOI (multiplicity of infection) of virus added to wells for 1 h, followed by the addition of 800 μL of 2% FBS Ham’s F12. Cells were incubated for 1 week, then stained for viral antigen by fluorescent microscopy as described above.

Vero E6 and PMVEC cells were plated onto 24-well plates coated with gelatin and incubated to confluence. Cells were inoculated with 0.1 MOI of MAPV for 1 h in 200 uL at 37 °C. Inocula were removed and the monolayers washed once in PBS. One milliliter of 2% FBS Ham’s F12 medium was added to each well. One hour later, 140 μL of supernatant was collected for RNA extraction (QiaAmp Viral RNA kit, Qiagen). The remaining medium was removed and discarded and cellular RNA was extracted from the monolayer (RNEasy kit, Qiagen). Additional samples were collected on days 3, 6, 9 and 12; on day 12 uninoculated samples were also collected (negative control). One-step PCR was performed in duplicate using an RNA Virus Master kit (Roche) with 1 uL of RNA, forward (5′ ATCAGGTTCAAGCCCTGTTG) and reverse (5′ AGGCAACTGGCAGATCTTGT) primers, and probe (5′ FAM-TGATTCACAGCCTCCTTTCC-BHQ1a) for 40 cycles according to manufacturer’s instructions (LightCycler 96, Roche, Pleasanton, CA, USA). TCID_50_ equivalents were determined by performing a log_10_ dilution of RNA generated from MAPV stock to estimate vRNA abundance in samples.

## 3. Results

### 3.1. Maporal Virus Persistently Infects Deer Mice

**Figure 1:**
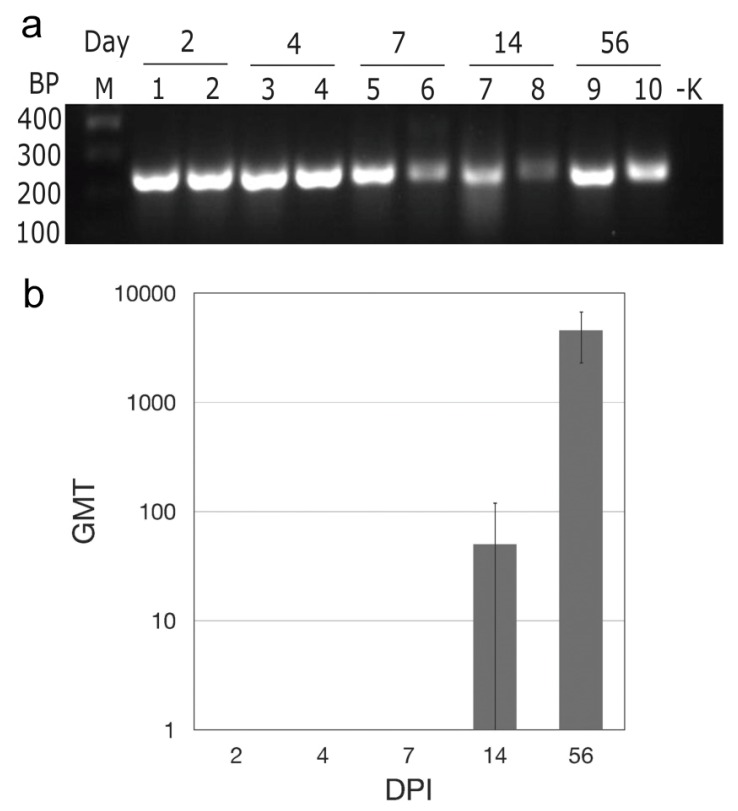
Maporal virus (MAPV) infects deer mice. After subcutaneous inoculation in the hindquarter, MAPV RNA was detected in the lungs two days later and persisted though at least 56 days; M marker, BP base pairs, -K negative control (**a**). Antibodies to MAPV nucleocapsid appeared as early as 14 days PI and increased in titer by day 56 (**b**).

In the initial experiment, we inoculated deer mice with 10^4^ TCID_50_ MAPV (subcutaneous, right hindquarters) and euthanized two each on days 2, 4, 7, 14, and 56 postinoculation to determine susceptibility and kinetics of infection in the lungs, a principal target organ of New World hantaviruses. None of the deer mice exhibited conspicuous signs of disease, similar to SNV and ANDV infection of deer mice [16,18,21]. PCR amplification detected MAPV sequences in all deer mice from each time point (Figure 1a), suggesting persistent infection. Attempts to isolate MAPV in Vero E6 cells were not successful. ELISA specific for nucleocapsid antigen showed that one deer mouse had seroconverted on day 14, as had both deer mice euthanized on day 56 (Figure 1b). None of the other deer mice had antibodies by ELISA. Neutralizing antibodies were only detected in the deer mice euthanized on day 56, with titers of 320 for each.

In a second experiment to determine tissue distribution of MAPV, five male deer mice were inoculated and euthanized 14 days PI. Viral RNA was detected by PCR in lungs, heart, spleen, kidney, liver and salivary glands of all five deer mice, a disseminated pattern similar to that found in some, but not all, deer mice persistently infected with SNV [17].

### 3.2. Histopathology and Immunohistochemistry

**Figure 2:**
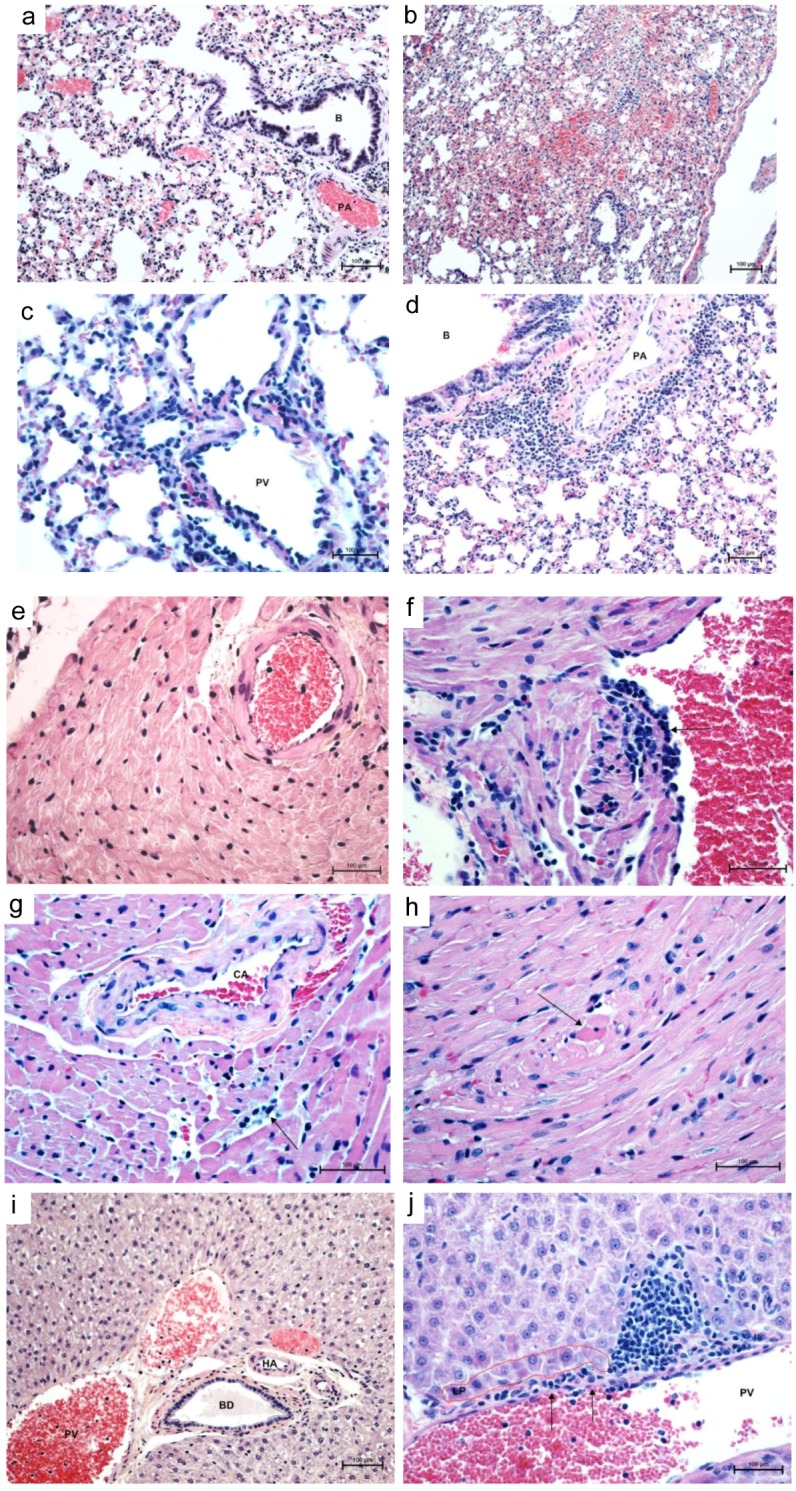
Histopathology of cardiopulmonary phase of MAPV infection in deer mice. Control lung (day 14) shows clear airways (bronchiole, B) and air spaces, normal vessels (pulmonary artery, PA) and normal thickness of interstitium (**a**, 200×). Lungs 14 days PI show alveolar hemorrhage, edema, and mild interstitial pneumonia (**b**, 100×). Lungs at 14 days PI show prominent perivascular (portal vein, PV, and artery, PA) neutrophilic infiltrates with mild interstitial pneumonia (**c**, 400×; **d**, 200×). Control heart shows a normal coronary artery and surrounding myocardium (**e**, 200×). Left atrium 7 days PI shows endothelial hypertrophy and hyperplasia with lymphocyte infiltration into subjacent myocardium with sarcoplasmic hyalinization and vacuolation of sarcoplasm with loss of cross striations (**f**, 400×). At 56 days PI, coronary artery (CA) with hypertrophic endothelium and perivascular edema, interstitial lymphocytic myocarditis and cardimyocyte degeneration/necrosis (**g**, 400×) and necrotic cardiomyocytes appear rounded up with hyperesinophilic sarcoplasm and pyknotic nuclei (**h**, 400×). Control liver shows intact limiting plate around normal portal vessels, hepatic artery (HA) and bile ductile (BD) (**i**, 200×). Focal disruption of portal vein (PV) endothelium (arrows) with lymphocytes percolating the limiting plate (LP) into the surrounding parenchyma mixed with a few neutrophils (**j**, 400×). Stain, hematoxylin-eosin.

At necropsy, organs of all deer mice appeared normal upon gross examination. Histopathologic lesions were detected in the lungs, liver, heart, and salivary glands of all infected animals to varying degrees (Figure 2, Table 1). At days 2, 4, and 56 PI, at least one infected deer mouse showed congestion of alveolar capillaries with multifocal pulmonary hemorrhages and minimal perivascular infiltrates of mixed inflammatory cells, predominantly neutrophils. Variable amounts of proteinaceous edema fluid filled small numbers of alveoli with occasional suspended macrophages. Severity of the lesions peaked at day 7 PI, especially in the lungs, heart, and liver. Peribronchiolar and perivascular neutrophilic infiltrates were prominent with minimal-to-mild interstitial pneumonia at 7–14 days PI. During the same time frame, the liver showed periportal infiltrates of mixed inflammatory cells, predominantly lymphocytes. Lesions in the heart were detected between 7 and 56 days PI and consisted of individual cardiomyocyte degeneration and mild lymphocytic interstitial myocarditis in the vicinity of affected cardiomyocytes. Occasionally, there was slight hypertrophy and hyperplasia of the endothelial lining of the left atrial endocardium and coronary vessels with subendothelial lymphocytic infiltrates into subjacent myocardium 7 days PI.

At 56 days PI, there were scattered necrotic/apoptotic cardiomyocytes characterized by rounding of a fragment of cardiac muscle fiber, which appeared hyalinized with loss of cross striations and vacuolation of sarcoplasm. Single-cell apoptosis was also detected in mucous salivary glands with minimal periductular infiltrates of lymphocytes. The spleen showed minimal to mild lymphoid hyperplasia, but no significant lymphocytolysis or other significant lesions. Apart from minimal interstitial infiltrates of lymphocytes and multifocal glomerular congestion, kidneys did not show other significant histologic lesions.

Immunohistochemical staining revealed no conspicuous immunoreactivity in any of the organs examined, similar to what is observed in ANDV-infected deer mice [21].

### 3.3. Gene Expression Profiling

**Figure 3:**
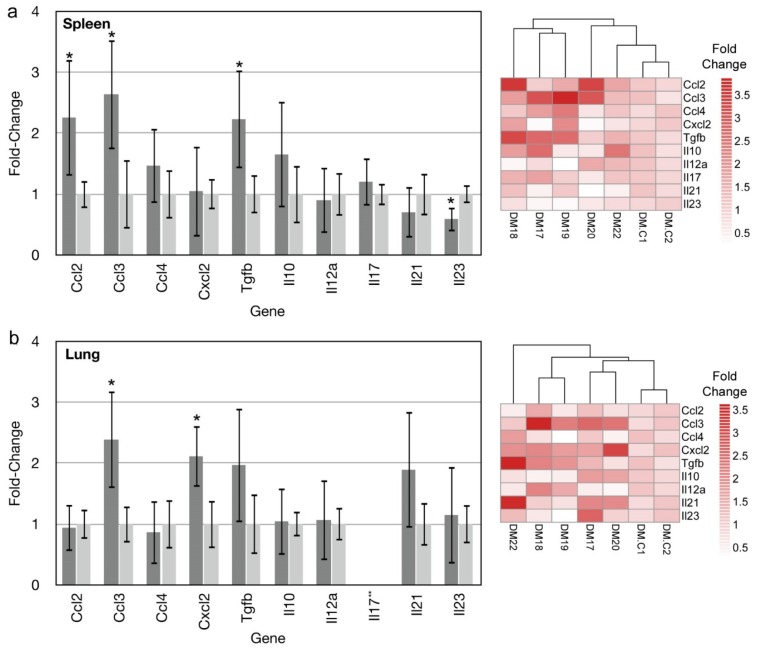
Cytokine and chemokine gene expression in spleens and lungs of MAPV-infected deer mice. Fourteen days post infection, spleens and lungs of 5 deer mice were examined for the expression of 10 genes by real-time PCR. *Ccl2*, *Ccl3* and *Tgfb* were elevated in spleens of infected deer mice (dark gray) compared to uninfected deer mice (light gray), whereas *Il23* expression was repressed. Error bars represent 95% confidence intervals and those denoted by * are statistically different from the uninfected control. Heat map indicates fold-change of individual deer mice used in generating the graph of infected deer mice (DM17–DM22) and uninfected controls (DM.C1, DM.C2) (**a**). In lungs, only *Ccl3* and *Cxcl2* were elevated. Heat map indicates fold-change of individual deer mice used in generating the graph of infected deer mice (DM17–DM22) and uninfected controls (DM.C1, DM.C2). **Il17 was not detected in either infected or uninfected lung samples (**b**).

Infection of deer mice with SNV or ANDV results in differential expression of many genes. We chose 10 of these genes for examination of lungs and spleens of MAPV-infected deer mice euthanized 14 days PI, a time point where many differences exist between ANDV- and SNV-infected deer mice [18,21], to determine whether MAPV infection more closely resembles ANDV or SNV infection. In spleens, *Ccl2*, *Ccl3* and *Tgfb* transcripts were significantly elevated, whereas *Il23* was significantly downregulated (Figure 3a). Spleen cluster analysis grouped three of the five deer mice (DM15, DM17, DM19) whereas one infected deer mouse (DM22) clustered near the two uninfected controls. In the lungs, *Ccl3* and *Cxcl2* were significantly elevated and no genes were downregulated (Figure 3b). One gene, *Il17* was not detected in the lungs of MAPV-infected or uninfected control deer mice. In contrast to the spleen data, DM22 clustered most distantly from the other four infected deer mice, as well as the two uninfected controls, and had the most abundant expression of *Il21* and *Tgfb*.

### 3.4. Maporal Virus Infects Deer Mouse Pulmonary Cells

**Figure 4:**
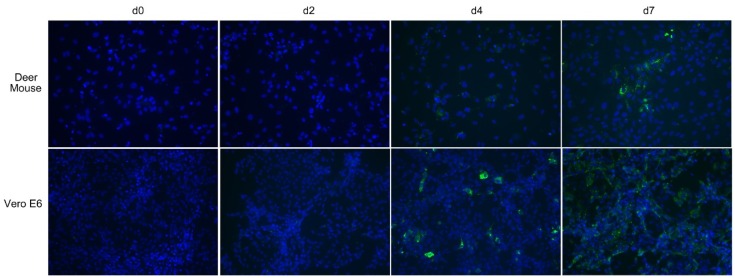
Maporal virus infects deer mouse cells. Deer mouse pulmonary microvascular endothelial cells (PMVEC) or Vero E6 cells were inoculated with 0.1 MOI of MAPV. On days 2, 4 and 7 cells from each were fixed and stained with rabbit antibody specific to nucleocapsid and detected with a mouse anti-rabbit IgG-FITC conjugate (green). Slides were mounted with DAPI to identify nuclei (blue). Viral antigen was detected in some deer mouse PMVEC on day 4 but substantially more cells by day 7, and with punctate characteristics. Virus was detected in few Vero E6 cells on day 2, but on day 4 punctate cells were readily observed. By day 7, many more cells were infected but the pattern was more diffuse and less puntate.

We cultured deer mouse pulmonary cells in endothelial cell medium to generate a primary cell culture to test susceptibility of the cells to MAPV. For in vitro infection experiments, deer mouse pulmonary microvascular endothelial cells (PMVEC) or Vero E6 cells were inoculated with MAPV, incubated and stained for viral antigen on days 2, 4, and 7, and examined by fluorescent microscopy. Microscopically, deer mouse PMVEC had substantially larger cytoplasmic area compared to Vero E6 (Figure 4), which are of epithelial origin. Viral antigen was not detected in day 2 infected deer mouse PMVEC, but on day 4 a few cells were antigen positive and on day 7 many more cells had detectable punctate antigen, including clusters of neighboring cells suggestive of lateral spread of the virus (Figure 4). Antigen was detected in some Vero E6 cells on day 2, but by day 4 punctate antigen was detected in many cells. By day 7, antigen detection was more widespread; however, it was diffuse instead of punctate. Detection of MAPV RNA from culture supernatants was equivocal except for day 7 Vero, where it was >10^3^ S segment copies/mL.

### 3.5. Maporal Virus RNA Accumulates in the Cellular Fraction of Deer Mouse PMVEC

**Figure 5:**
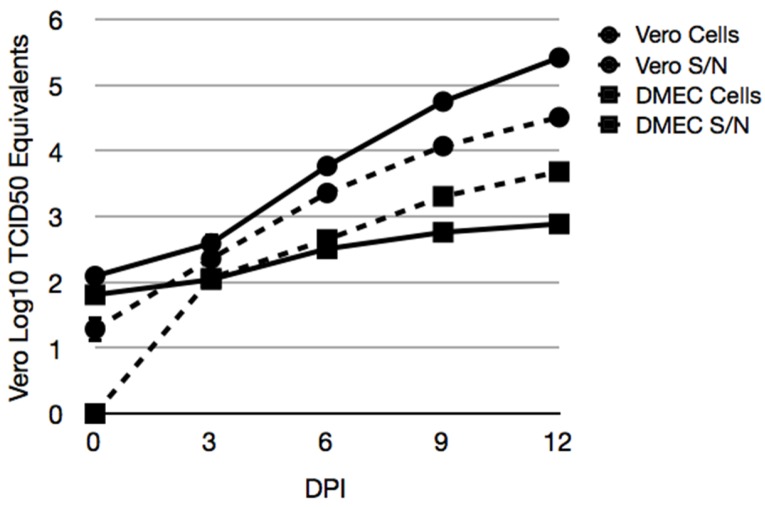
Accumulation of MAPV RNA in cellular fractions of infected deer mouse PMVEC. Vero E6 (circles) and deer mouse PMVEC (squares) were infected with MAPV to examine viral RNA in cells (hatched lines) and supernatants (solid lines) by real-time PCR. Cells were inoculated with 0.1 MOI of MAPV for 1 h, followed by removal of inoculum, 1× wash in PBS and addition of 2% FBS Ham’s F12 medium. The day 0 samples were collected 1 h later. The remaining samples were collected on days 3, 6, 9 and 12 dpi. RNA copies were determined relative to tissue culture infectious dose (TCID_50_) equivalents.

Vero E6 and deer mouse PMVEC were inoculated with 0.1 MOI of MAPV to quantify viral RNA levels by real-time PCR in supernatants and cells (Figure 5). vRNA levels were substantially higher in Vero E6 cells than in PMVEC. The amount of viral RNA in the Vero E6 supernatant exceeded the amount in its cellular fractions. However, infection of PMVEC led to a greater accumulation of viral RNA in its cellular fraction by day 6. By day 12, more than 5 logs (TCID_50_ equivalents) of vRNA was detected in Vero E6 supernatants, but less than 3 logs were detected in PMVEC supernatants. In contrast, about 4.5 logs of vRNA was detected in the Vero E6 cellular fraction and about 3.8 logs in the PMVEC cellular fraction.

## 4. Discussion

Several reservoir host infection models have been developed for studying pathogenic hantavirus infections; Old World Seoul virus infection of Norway rats (*Rattus norvegicus*) and Puumala virus infection of bank voles (*Myodes glareolus*), and two New World viruses, Black Creek Canal virus infection of cotton rats (*Sigmodon hispidus*) and SNV infection of deer mice [16,32,33,34,35]. Deer mice can also be infected with ANDV, which is naturally hosted by the long-tailed pygmy rice rat (*Oligoryzomys longicaudatus*), but it is cleared after several weeks [21]. Pathogenic Old World hantaviruses require ABSL-3 containment, whereas New World human pathogenic hantaviruses require ABSL-4 containment [22], which restricts the number of institutions where such work can be performed and substantially limits understanding of how New World viruses evade sterilizing immune responses in reservoir hosts, a critical feature of zoonotic virus ecology.

As natural reservoir hosts of SNV, deer mice remain persistently infected [17], perhaps for life [13,27,36,37,38]. Deer mice are one of the only New World reservoir hosts that have been colonized for hantavirus research. Therefore, we sought to develop an experimental infection model of this species that can be manipulated under ABSL-3 containment. We chose Maporal virus for our model because (a) it causes an HCPS-like disease in Syrian hamsters [25] and (b) it is not known to cause human disease and can, thus, be manipulated in ABSL-3 environments.

In our initial infection experiment to determine susceptibility, we euthanized one male and one female at various time points and detected viral RNA by PCR in the lungs of all deer mice. A previously developed ELISA using a highly conserved B cell epitope of Sin Nombre virus nucleocapsid [26,39] was used to determine antibody titers. Antibodies were detected in one of two deer mice euthanized on day 14 (male, titer of 200), and both deer mice euthanized on day 56 (titers of 3200 for the male and 6400 for the female). Only the deer mice euthanized on day 56 had detectable neutralizing antibodies, with titers of 320 for each. In SNV infection, low-titered ELISA antibodies to nucleocapsid are detected on day 14 in some deer mice [18], but in ANDV infection all deer mice produce antibodies by day 14, some with modestly high titers [20,21]. Although slower to develop, deer mice mount a more robust neutralizing antibody response to SNV (titer range of 160–5620) [16,17] than to ANDV (range 10–80) [21]. Thus, the deer mouse IgG response to MAPV is more similar to SNV than to ANDV.

Experimental infection of deer mice with SNV results in detection of viral RNA in many tissues [16]. In this study, deer mice infected with MAPV also displayed a disseminated infection pattern at 14 days PI, with virus detected in all organs of all deer mice examined: lung, heart, spleen, kidney, liver, and salivary gland. The tissue distribution of ANDV in deer mice is less well characterized, but viral RNA can be detected in lung, heart and spleen to day 21; however, virus is generally cleared by day 56 [21].

Pathophysiologically, the deer mouse model of MAPV infection recapitulates some of the histologic features of the cardiopulmonary phase of HCPS in humans and hamsters. The lesions in the lungs, heart, and liver are not severe enough to precipitate a clinical disease and can be detected in infected deer mice for 8 weeks PI. Vascular leakage plus acute interstitial pneumonia and single cardiomyocyte necrosis are central to pathogenesis of MAPV infection in deer mice and resembles ANDV infection of hamsters [40]. However, it is interesting that histologic lesions are present in major organ systems despite general lack of unequivocal immunohistochemical reactivity in any of the involved organs. Previous studies of SNV infection of deer mice encountered such an important paradox with one major difference that the current model shows pathology in cardiopulmonary organs [41]. Deer mice experimentally infected with SNV also exhibit mild histopathologic changes with the presence of viral antigen in the same infected organs [16]; however, ANDV fails to induce histopathology in deer mice and viral antigen is not detected in tissues [21]. Thus, MAPV infection of deer mice shares histopathologic similarities with SNV infection but without detection of antigen, similar to ANDV infection.

Two weeks after experimental infection of deer mice with SNV, only low to modest levels of certain immune genes that rarely exceed 4-fold elevation occurs [18]. In contrast, two weeks after infection with ANDV a robust immune gene expression pattern occurs that is qualitatively and quantitatively distinct, with some genes elevated more than 20-fold, which may account for clearance of ANDV but not SNV [21]. We examined the expression of 10 cytokine and chemokine genes in MAPV-infected deer mice that have different patterns and levels in SNV and ANDV infected deer mice. MAPV elicits an expression pattern more similar to SNV than to ANDV in terms of expression levels. In the spleen, *Ccl2*, *Ccl3*, and *Tgfb* levels were 2- to 3-fold above that of uninfected control deer mice. Splenic *Ccl2* expression is elevated in SNV-infected deer mice but not in ANDV-infected deer mice. *Ccl3* expression in ANDV spleens is about twice that of SNV and MAPV, and *Tgfb* expression is about four times greater in ANDV spleens than in SNV- or MAPV-infected spleens. *Il23* expression is substantially downregulated in ANDV- [21] and MAPV-infected spleens, but less so in MAPV infection. Its levels are unchanged in SNV infected deer mouse spleens [18], thus *Il23* expression during MAPV infection resembles that of ANDV infection.

In lungs, only *Ccl3* and *Cxcl2* expression were elevated in MAPV infected deer mice. *Ccl3* is elevated in response to ANDV infection, but not SNV infection, whereas *Cxcl2* levels are similarly elevated with MAPV and SNV infection, but much higher in ANDV infection [18,21]. *Tgfb* expression was elevated in MAPV-infected deer mice, but because of substantial variation between animals it was not statistically different. *Tgfb* is statistically elevated in lungs of both SNV and ANDV infected deer mice two weeks after infection, but substantially higher in ANDV infection. In the present work, deer mouse DM20 accounted for much of the *Tgfb* variation, with a fold-change of 0.76; its removal from the analysis resulted in statistically elevated *Tgfb* expression. Thus, although there are several differences in expression patterns of these genes, the data suggest that MAPV infection of deer mice more closely resembles that of SNV than ANDV. We have previously reported large variation in gene expression in deer mice infected with SNV [18] that we attribute, in part, to their outbred status, and these results are consistent with those findings.

We used endothelial cell medium containing a cocktail of endothelial cell growth factors to cultivate PMVEC from a 7 day old deer mouse. Immunofluorescence assay (IFA) microscopy suggested that MAPV may directly transmit to adjacent PMVEC cells (Figure 4). The faster and greater replication of MAPV in Vero E6 is likely due to the defective type I interferon (IFN) response in these cells that, presumably, is intact in the deer mouse PMVEC and likely retards virus replication. However, it is also possible that the deer mouse PMVEC culture contains other cell types that are not susceptible to MAPV. Although Vero E6 is deficient in its type I IFN response, hantaviruses can induce type III IFNλ (IL-29) production in Vero E6 about a week after infection [42], thus it is possible the diffuse appearance of the day 7 Vero E6 cells may be caused by the presence of IFNλ stimulation of the innate response. When cellular vs extracellular (i.e., supernatants) levels of viral RNA were examined, Vero cell supernatants had substantially more vRNA than did its cellular fraction (Figure 5). However, for the deer mouse PMVEC, the cellular fraction had more vRNA than did the supernatants from day 6 and beyond. This also suggests that MAPV infection of deer mouse PMVEC may occur directly cell to cell, and that virus cellular shedding may be a minor component in reservoir host endothelial cells. Further studies are required to determine the mechanisms that account for this observation.

We have determined that deer mice are experimentally susceptible to Maporal virus and that infection resembles that of SNV, although with some similarities to ANDV (Table 2). In total, 15 deer mice were inoculated and all became infected. It is the third New World hantavirus that has been shown to experimentally infect deer mice without precipitating overt clinical disease. However, MAPV induces mild histopathologic alterations reminiscent of HCPS in humans and which appear to be more substantial than SNV infection of deer mice. This system will facilitate investigations of a New World reservoir host response to hantaviruses without requirement for ABSL-4 containment.

MAPV causes an HCPS-like disease in Syrian hamsters, thus it should be possible to directly compare the host responses of a reservoir host species to that of a pathology model using the same virus. The ability to generate deer mouse primary cell lines that support replication of hantaviruses could also facilitate examination of homologous hantavirus (SNV) strategies for manipulation of the host cell response, as well as identification of differences between heterologous hantaviruses. Evidence has recently accumulated suggesting hantaviruses manipulate the antiviral response of infected cells [43,44,45,46]. However, much of this work has been performed in immortalized human and nonhuman primate cell lines, and with viral genes cloned into high-expression plasmids that probably do not reflect nominal levels that occur in infected cells. Considering that MAPV, SNV and ANDV can infect deer mice, it is likely that these cells are also susceptible to these viruses, and probably other New World hantaviruses. Together, this could reveal the mechanisms that have evolved in hantaviruses that lead to persistent infection of the reservoir hosts, and how they may contribute to pathogenesis in humans.

## Figures and Tables

**Table 1:** Histopathology of deer mice infected with Maporal virus.

Mouse #	DPI	Lungs				Heart	
		Cong./Ede	MIAH	IP	PBLH	CD/apop	LIM
DM 1	2	+	+	-	-	-	+
DM 2	2	++	+	+/−	-	+/−	+/−
DM 3	4	++	+	+/−	+	+/−	+/−
DM 4	4	+	-	-	-	+/−	+/−
DM 5	7	++	-	+	+	+/−	+
DM 6	7	++	+/−	+	++	+	++
DM 7	14	+/−	+/−	-	+/−	-	+/−
DM 8	14	+/−	++	+/−	-	-	+/−
DM 9	56	+	+/−	+/−	+/−	-	+/−
DM 10	56	+	+	-	-	-	-

-, Negative (no histologic lesions detected in the sections examined); +/−, Minimal inflammation or degeneration/apoptosis; +, Mild inflammation or degeneration; ++, Moderate inflammation or degeneration; Cong. = congestion; Ede = edema; MIAH = multifocal intraalveolar hemorrhage; IP = interstitial pneumonia; PBLH = peribronchiolar lymphoid hyperplasia; CD = cardiomyocyte degeneration; Apop = apoptosis; LIM = lymphocytic interstitial myocarditis.

**Table 2:** Comparison of MAPV, Sin Nombre virus (SNV), and Andes virus (ANDV) infection of deer mice. Traits in boldface reflect those most similar to SNV or ANDV infection of deer mice.

Feature	MAPV	SNV	ANDV
Clinical Disease	None	None	None
Outcome	Persistence	Persistence	Clearance
Nucleocapsid Ab day 14	Some	Some	All
Neutralizing Ab titer	High	High	Low
Cytokine gene expression	Low	Low	High
Histopathology	Minimal-Mild	Minimal	None
Nucleocapsid IHC	None	Low	None
